# The Quarantine Question

**Published:** 1897-05

**Authors:** 


THE QUARANTINE QUESTION.—The following letter, which appeared in the *Texas Medical News* for April, '97, is reproduced as a matter of general interest to our readers.

QUARANTINE DEPARTMENT,
OFFICE OF STATE HEALTH OFFICER,
AUSTIN, TEX., April 10, 1897.

*Editors Texas Medical News:*

In the matter of the memorial from the Texas State Medical Association requesting the legislature to transfer the expense of quarantine to the federal government, the committee, Drs. J. W. McLaughlin, A. N. Denton, W. R. Blailock and J. C. Loggins, appointed to present said memorial, in their zeal to perform the full measure of the duties assigned them, have probably gone too far, and made statements that are incorrect. They say that Texas has not the facilities for treating infected vessels and cargoes, nor of taking care of passengers and crews of infected vessels," that "the quarantine practice of the State is to refuse admission to infected vessels."

When men speak for themselves, they should be guarded in statements that affect the character of others, but when they speak for a great organization on a question that involves the honor and reputation of members of that organization, they should, I think, be absolutely accurate.

Fortunately, there are members of the present legislature who have visited Galveston, and who know that we there have a splendid plant for treating infected vessels, and that infected vessels have been there treated nearly every month for eight or ten years by the most improved methods of modern maritime sanitation. They also know that we have facilities for caring for passengers and crews, unsurpassed by any quarantine station in the world.

If those advocates of a centralized government who can see no good in our State quarantine entertain doubts as to the correctness of my statements, I respectfully invite them to visit the station at Galveston, and be their own witnesses.

I do not think that the honorable gentlemen who signed that petition intended to create a false impression, or desired to do injustice to the State health authorities; but a wrong has been done, and it ought to be righted.

While on this subject, permit me to say again that I favor a State board of health, and will gladly do all in my power to secure the necessary laws for its creation, but I believe that Texas is great enough, strong and rich enough, to take care of herself in health matters, without the intervention or aid of the Federal government. Yours truly,

R. M. Swearingen, M. D.

To the above Dr. McLaughlin, editor of the *News*, chairman of the committee to memoralize the legislature, and author of the "memorial," replied at great length, begging the question entirely. He quibbles over the construction of the words "infected vessels," and claims that while the committee meant vessels having quarantinable diseases on board, Swearingen means vessels in good sanitary condition (non-infected) but coming from points south of 25 degrees; interdicted points. Just by what process of reasoning he arrives at the conclusion that Swearingen does not mean "infected vessels" when he says "infected vessels," is beyond my ken.

The charge is made in the memorial, that "Texas has not the facilities for disinfecting infected vessels; nor of caring for passengers, crews and cargo of such vessels." This assertion is made without qualification; yet in his reply to Dr. Swearingen's protest, Dr. McLaughlin says "the committee were informed that two stations—Galveston and Sabine Pass, are provided with modern facilities for ship disinfection," while at all other stations—(where no infected vessels—but clean vessels from suspected infected ports arrive)—fumigation is done by burning sulphur.

Here we have the assertion laid before the legislature without qualification that "Texas has not the facilities for disinfecting infected vessels." Later—in effect, it is admitted that at the only two stations where really infected vessels arrive, the committee knew that there *are* all modern facilities. It would seem that a spirit of fairness would have prompted a qualification of the assertion, at least, as regards Galveston and Sabine Pass. The assertion is, therefore, unjust and misleading, and ought not to be allowed to pass without protest. To say to the legislature, Texas has, practically, no quarantine—admitting meantime—later, after the damage is done—that she *has* modern facilities at the two chief ports,—is well calculated to create a sensation, in light of the fact that the several legislatures have appropriated in several years $15,000 to provide such facilities. It is a reflection upon the State Health Officer. Members will naturally ask—where has the $15,000 gone, if we have nothing to show for it?

The facts are, demonstrably, that at Sabine Pass there is a plant of the Holt system of maritime sanitation—the most modern,—in general use, for which the State paid in 1889 $5,000. At Galveston there is a disinfecting warehouse on Pelican Spit, opposite the city, with capacity to receive the cargo of several large vessels for fumigation, a Holt system of sanitation by which the gas is driven by steam fans; steam chambers in which clothing and other articles are subjected to superheated medicated steam, drying chambers for hot air;—and while the clothing and baggage of immigrants are being thus disinfected—the immigrants, stripped, are subjected to a salt shower bath; made to run the gauntlet of a shower the length of a hall some 25 feet long; beds and rooms for the accommodation of at least one hundred persons. In addition to this there is a steam tug—the Hygiea—built for the purpose, and costing $17,000. Dr. Swearingen, it will be seen, invites the gentlemen who have made the assertion that "Texas has no facilities for treating infected vessels, nor for the detention and care of passengers and crews," to visit Galveston and be their own witness. The question thus resolves itself into one of veracity.

In Dr. McLaughlin's reply he says that "if it is a fact that infected vessels are sent by the State quarantine authorities to the marine hospital stations to be treated, Dr. Swearingen is in the pillory he erected for the committee; if it is not the practice to send infected vessels to federal stations for treatment—an apology is due Dr. Swearingen." The JOURNAL is informed by State Health Officer Swearingen that never, within the last eight years, has any vessel been referred to any federal station for treatment, except in one single instance; and that was several years ago, and before the new warehouse at Galveston was built, and it was at the urgent solicitation of officers and passengers—(who had the option) and who hoped for a shorter period of detention. To a disinterested party it would seem that an apology is in order.

The Federal law of 1893 makes it the duty of the Supervising Surgeon-General of the Marine Hospital Service, *if, in his opinion*, from any cause whatever, any State quarantine is inefficient—to take charge of that station without more ado. That the Supervising Surgeon-General of the M. H. S. has *not* taken charge of any Texas quarantine convicts him of neglect of duty if the assertions of our memorialists are true; that he has not done so is evidence that he "begs to differ with Brother McLaughlin," and does not think that "Texas has no facilities for treating infected vessels." His non-action utterly refutes the charges. If further proof were needed we would quote the report of Surgeon Sawtelle, a Marine Hospital Inspector, who annually inspects all State quarantine Stations. (See report Surgeon-General, M. H. S., 1896.) Surgeon Sawtelle testifies to adequate facilities at the Texas quarantine, and to the efficiency of the State service.

